# Cross-Modality Medical Image Segmentation via Enhanced Feature Alignment and Cross Pseudo Supervision Learning

**DOI:** 10.3390/diagnostics14161751

**Published:** 2024-08-12

**Authors:** Mingjing Yang, Zhicheng Wu, Hanyu Zheng, Liqin Huang, Wangbin Ding, Lin Pan, Lei Yin

**Affiliations:** 1 College of Physics and Information Engineering, Fuzhou University, Fuzhou 350108, China; yangmj5@fzu.edu.cn (M.Y.); a931048011@gmail.com (Z.W.); 231120030@fzu.edu.cn (H.Z.); hlq@fzu.edu.cn (L.H.); 2 School of Medical Imaging, Fujian Medical University, Fuzhou 350122, China; dingwangbin@fjmu.edu.cn; 3 The Departments of Radiology, Shengli Clinical Medical College of Fujian Medical University, Fuzhou 350001, China; 4 Fujian Provincial Hospital, Fuzhou 350001, China; 5 Fuzhou University Affiliated Provincial Hospital, Fuzhou 350001, China

**Keywords:** cross modality segmentation, unsupervised domain adaptation, feature alignment, cross pseudo supervision

## Abstract

Given the diversity of medical images, traditional image segmentation models face the issue of domain shift. Unsupervised domain adaptation (UDA) methods have emerged as a pivotal strategy for cross modality analysis. These methods typically utilize generative adversarial networks (GANs) for both image-level and feature-level domain adaptation through the transformation and reconstruction of images, assuming the features between domains are well-aligned. However, this assumption falters with significant gaps between different medical image modalities, such as MRI and CT. These gaps hinder the effective training of segmentation networks with cross-modality images and can lead to misleading training guidance and instability. To address these challenges, this paper introduces a novel approach comprising a cross-modality feature alignment sub-network and a cross pseudo supervised dual-stream segmentation sub-network. These components work together to bridge domain discrepancies more effectively and ensure a stable training environment. The feature alignment sub-network is designed for the bidirectional alignment of features between the source and target domains, incorporating a self-attention module to aid in learning structurally consistent and relevant information. The segmentation sub-network leverages an enhanced cross-pseudo-supervised loss to harmonize the output of the two segmentation networks, assessing pseudo-distances between domains to improve the pseudo-label quality and thus enhancing the overall learning efficiency of the framework. This method’s success is demonstrated by notable advancements in segmentation precision across target domains for abdomen and brain tasks.

## 1. Introduction

**Figure 1:**
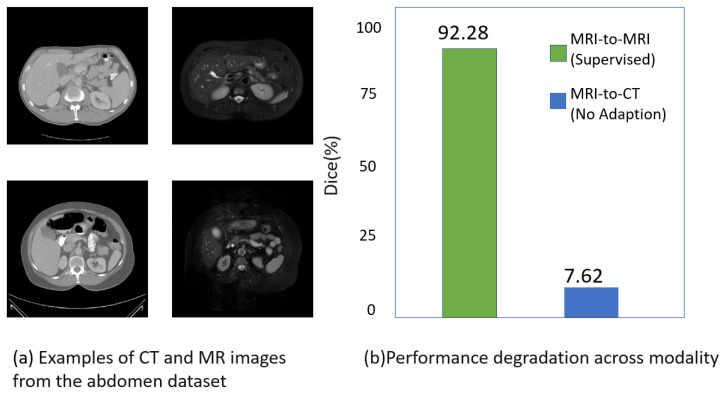
Illustration of the cross-modal medical image challenge. (**a**) Comparison of the modal gap between CT and MR images in abdominal images. The liver CT image is on the left and the MRI image is on the right. (**b**) Comparison of the models trained on abdominal MRI images, which were evaluated on MRI and CT images using the Dice score and are denoted as “supervised” and “no adaptation”, respectively.

The commendable accomplishments of deep convolutional neural networks (CNNs) in image segmentation have been extensively documented [1,2]. The anticipation is that deep learning models should adeptly handle the diverse medical images originating from various imaging modalities, scanning protocols, or demographic factors. However, in practical healthcare applications, a notable disparity often arises between the data used for training and that encountered during testing, which is attributed to differences in acquisition parameters or imaging modalities [3]. A considerable degradation in the performance of the trained model may be caused by this gap. As illustrated in Figure 1, differences in the image data obtained from abdominal images using magnetic resonance imaging (T2-SPIR MRI) and computed tomography (CT) modalities are evident. This phenomenon, where algorithm performance is degraded due to differences in data distribution during cross-modality image processing tasks, is referred to by researchers as the domain shift. Therefore, the model’s capability to generalize across various domain shifts needs to be enhanced.

Models trained on the source domain cannot be directly migrated to the target domain (e.g., MR to CT) for effective segmentation due to the presence of significant domain offsets. To mitigate cross-modality performance degradation, a plain and intuitive approach is to use labeled images on the target domain for fine tuning. However, this approach necessitates extensive target domain annotation, and pixel-level annotation on medical images is found to be very labor-intensive and time-consuming.

Unsupervised domain adaptation (UDA) techniques perform a core idea to deal with cross-modal analysis [4,5]. In the UDA setting, given a labeled source domain dataset and an unlabeled target domain dataset, the goal is to train a robust model capable of achieving high segmentation performance on the target domain. Currently, most advanced UDA segmentation methods are at the image level [6,7,8], feature level [9,10,11], or joint image and feature level [12,13], with adversarial learning mechanisms being used to train and optimize the model. The fundamental approach at the image level involves using GANs to generate source domain images into the target domain. These generated images are then utilized to train models for the target domain, aiming to enhance the performance of the target domain model. On the other hand, feature-level methods focus on mapping data from both source and target domains to a domain-invariant space, which are then subsequently applied to downstream tasks such as image segmentation. Among them, a pivotal work is CFDnet [14], which focuses on minimizing the distance of distribution feature functions for feature alignment.

In the context of cross-modal image generation, these methods commonly face the following challenges: the quality of generated images in the target domain significantly influences the performance of downstream segmentation networks. If features fail to align effectively, it may lead to noticeable fluctuations during network training, making the training process complex and susceptible to the collapse phenomenon. Additionally, such methods encounter issues of subpar individual segmentation results in the target domain, contributing to a substantial variance in performance across multiple model training iterations. The fundamental cause of these problems can be primarily attributed to ineffective feature alignment, resulting in the inability of transformed cross-modal images to be effectively used for training segmentation networks.

**Figure 2:**
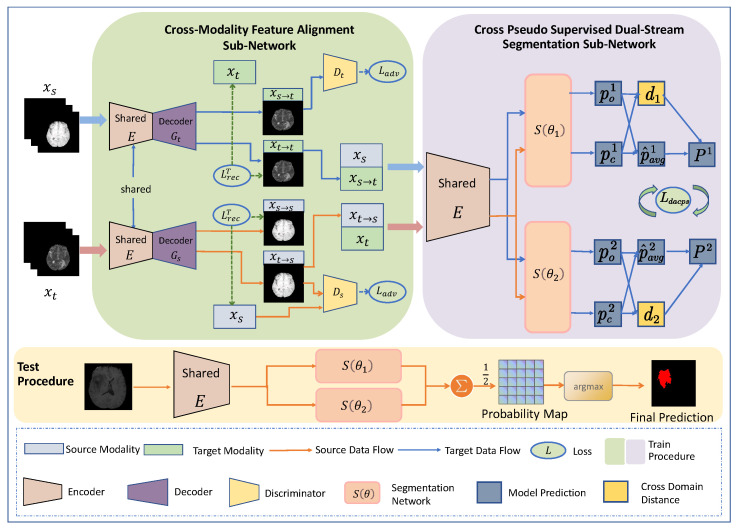
An overview of our proposed approach. The whole network consists of a cross-modal, shared common encoder (*E*), two domain-specific discriminators (Ds, Dt), two domain-specific private decoders (Gs, Gt), and two segmentation networks (S(θ)). Among them, the shared encoder and the domain-specific decoder are used for feature alignment. The cross-pseudo-supervised, dual-stream segmentation sub-network consists of two segmentation networks ($S(\theta_{1})$, $S(\theta_{2})$) and a distance-aware, pseudo-supervision module. Self-attention modules are added to the last convolutional layer of the encoder, which is not shown in the diagram. In the inference phase, we only need to test the images from the target domain. We took the ensemble of the two networks as the final prediction.

In addressing the challenges outlined, this study introduces a novel unsupervised domain adaptation image segmentation framework, as illustrated in Figure 2. The primary function of the feature alignment sub-network is to effectively align the distributions of the source and target domains, performing image generation and reconstruction tasks. Building upon the network introduced by Han et al. [13], a self-attention module [15] is incorporated to effectively model both long-range and multi-level dependencies among image regions, allowing the model to capture information pertinent to geometric and structural consistency more accurately. Furthermore, to reciprocally promote feature alignment and facilitate the training of downstream segmentation networks inspired by Chen et al. [16], a dual-stream segmentation sub-network is proposed with consistent architectures, wherein the two streams are initialized differently. The outputs of both streams are constrained by a cross-pseudo-supervised loss [16], encouraging consistency in the outputs of the dual-stream network to promote cross-modal feature alignment. Given the significant differences between our task and traditional single-modal multicenter scenarios, where there is considerable inter-modal variation, we introduce a cross-domain distance awareness module to enhance the quality of pseudo-labels and alleviate the pronounced fluctuations in segmentation loss. Our model represents a distinct paradigm from a segment anything model (SAM) [17], focusing on domain adaptation for medical image segmentation with full automation and minimal reliance on target domain labels, thus contrasting SAM’s semi-automated, generalization-based approach that necessitates broad dataset training and manual fine tuning.

Our innovative points are summarized as follows:

- We propose a novel, unsupervised domain adaptation framework for medical image segmentation, comprising a feature alignment sub-network and a pseudo-supervised, dual-stream segmentation sub-network. The feature alignment sub-network facilitates the alignment of features between the source and target domains, while the pseudo-supervised, dual-stream segmentation sub-network achieves segmentation of cross-modal images and promotes the learning process of the feature sub-network;
- For the first time, cross-pseudo-supervision is introduced to mitigate the impact of low-quality pseudo-labels, thereby insulating the segmentation network from fluctuations in the feature alignment sub-network;
- Given that medical images present clear structures and shapes, incorporating a self-attention module into the feature alignment sub-network significantly improves the learning process;
- Our method was evaluated on two challenging tasks, and the results indicate that it is increasingly approaching the performance of fully supervised models.

## 2. Related Works

### 2.1. Cross-Modality Medical Image Segmentation

Cross-modality medical image segmentation [18,19,20] involves the transfer and application of knowledge from a source domain to enhance segmentation in a target domain. Kang Li et al. [20] proposed an approach using online mutual knowledge distillation. Over years of development, researchers have proposed various types of methods to address cross-domain problems.

Domain adaptation technology has significantly advanced in addressing domain discrepancy issues such as cross-modality medical image segmentation. The journey began with Single-Source Domain Adaptation [5,21,22], focusing on transferring knowledge learned from one source domain to the target domain. This was followed by multi-source domain adaptation [23,24,25], which enhances the effectiveness of transfer learning by integrating data from multiple source domains to capture a broader set of domain knowledge. Pei et al. [23] proposed a framework that utilizes multi-level adversarial learning and a multi-model consistency loss to adapt features and transfer knowledge from multiple source domains to the target domain.

With the challenge of scarce source labels, semi-supervised domain adaptation methods [26,27,28] emerged, utilizing both limited labeled data and a large amount of unlabeled data for improved domain adaptation. Zhao et al. [26] introduced MT-UDA, a label-efficient framework where a student model, trained with limited source labels, learns from unlabeled data in two domains through two teacher models in a semi-supervised manner. This method distills intra-domain semantic knowledge and exploits inter-domain anatomical information, thereby improving cross-modality segmentation performance by integrating underlying knowledge and addressing source label scarcity.

Recently, source-free domain adaptation [29,30,31] has gained attention, where the aim is to achieve domain adaptation using only a pre-trained source domain model without access to source domain data. This approach faces the challenge of effective knowledge transfer without direct support from the source data. M. Bateson et al. [29] utilized a novel loss function that combines Shannon entropy and KL divergence to match class ratios with anatomical priors, and it is validated on cardiac, prostate, and intervertebral disk segmentation tasks.

The evolution of these technologies demonstrates the ongoing depth and innovation within the domain adaptation field, and it is aimed at overcoming data distribution differences and improving model generalization in new domains.

### 2.2. Single-Source Domain Adaptation

Single-Source Domain Adaptation, as explored in the works of Wang et al. [21], Saito et al. [4], and Long et al. [32], constitutes a vital aspect of transfer learning. It finds extensive applications in addressing performance degradation issues when the distribution of test data significantly deviates from that of the training data [33]. In the realm of medical images, domain adaptation holds even greater potential. Pei et al. [22] proposed an innovative unsupervised domain adaptation framework tailored for cross-modal heart image segmentation. Their approach segregates domain-invariant and domain-specific features for each domain, augmenting the representation of domain-invariant features through the incorporation of self-attentive modules and a zero-loss strategy. Ouyang et al. [34] introduced a data-efficient, unsupervised domain adaptation method that fuses variational, self-encoder-based features prior to matching with domain adversarial training. This hybrid approach effectively learns a shared domain-invariant hidden space. Building upon a self-training strategy and adversarial learning, Xie et al. [5] enhanced target-domain image segmentation performance. Chen et al. [8] leveraged generative adversarial networks to transform image appearances across modalities. Their approach incorporates deep collaborative image and feature alignment, and it is complemented by self-attentive mechanisms and knowledge distillation losses to elevate segmentation quality. Han et al. [13] devised a novel deep symmetric adaptation network for cross-modal medical image segmentation. The architecture comprises a segmentation sub-network and two symmetric source and target domain transformation sub-networks. These sub-networks implement a bi-directional alignment strategy by sharing an encoder and two private decoders. The use of original and transformed images for training the segmentation classifier effectively diminishes inter-domain differences, leading to improved segmentation performance. For cross-modality segmentation, the mainstream method is still used to perform feature alignment and to then apply the domain-invariant features to the training of the segmenter. Our network also includes these two parts.

### 2.3. Feature Alignment

The concept of feature alignment is a prevalent theme in various unsupervised domain adaptation (UDA) frameworks. Many UDA methods concentrate on aligning the distribution in the feature space by minimizing the distance metric between features extracted from the source and target domains. Adversarial learning is a prominent approach in this context [35,36]. Ganin et al. [36] leveraged adversarial training to obtain domain-invariant features between the encoder and decoder, resulting in the learning of domain-invariant representations. Liu et al. [37] introduced a semantic segmentation branch with a domain discriminator to address the domain gap at the context level. Han et al. [13] proposed a bidirectional alignment strategy that trains the segmentation classifier using both original and transformed images, effectively reducing inter-domain discrepancies. Inspired by Han et al. [13], we incorporate analogous ideas into our framework to enhance feature alignment.

### 2.4. Cross-Pseudo-Supervision

Cross-pseudo-supervision (CPS) was introduced by Chen et al. [16] in 2021. The fundamental concept involves employing two networks with identical structures but different initializations, imposing constraints to ensure that the outputs of both networks for the same sample exhibit similarity. Ouali et al. [38] extended the CPS framework to propose a cross-consistency training method for cross-modal, semi-supervised semantic segmentation. This method incorporates data enhancement and feature alignment techniques for images from different modalities. Ibrahim et al. [39] presented a self-correcting network approach aimed at enhancing the quality of pseudo-labeling and the robustness of the model. This is achieved by introducing a correction module and a confidence module to dynamically update pseudo labels and loss weights. Feng et al. [40] focused on improving the generalization ability of the model within the context of CPS. They introduced a dynamic thresholding strategy and a class-balancing strategy to effectively select high-quality pseudo labels and balance the difficulty differences between different classes. In our experiments, we observed that the semi-supervised approach of CPS proves effective in harnessing unlabeled data and facilitating the learning of feature-aligned networks. For unsupervised domain adaptation, which can be viewed as a special case of semi-supervised learning, CPS demonstrates its ability to enhance unsupervised domain adaptation capabilities.

## 3. Method

### 3.1. Datasets

In our study on cross-modality segmentation, we consider tasks involving abdominal organ segmentation and brain tumor segmentation due to their clinical significance and the challenges they present in terms of imaging modalities.

For abdominal organ segmentation, we utilized a comprehensive dataset that included 30 CT scans from the BTCV [41] challenge training set and 20 T2-SPIR MRI scans from the ISBI 2019 CHAOS challenge [42] training set. These datasets present a variety of volume sizes and resolutions: the BTCV, with slice thicknesses ranging from 2.5 mm to 5.0 mm; and the CHAOS with ISDs, varying from 5.5 mm to 9 mm and averaging at 7.84 mm, alongside x-y spacing that ranges from 1.36 mm to 1.89 mm, averaging at 1.61 mm. To streamline consistency, we cropped the CT images, initially encompassing 11 organs, to focus solely on the abdominal area, including the liver, both kidneys, and the spleen, and we retained the annotations of these four organs from the BTCV dataset.

The brain tumor segmentation, derived from the Brats2018 Brain Tumor challenge, consisted of 75 instances pf patient data from the LGG category, including the following four modalities: T1, T1CE, T2, and FLAIR. Targeting whole tumor segmentation, domain adaptation experiments were conducted with T2 as the source domain and the other three modalities as the target domain. Across all datasets, in line with SIFA [8] and SymDA [13], 80% of patient data were randomly selected for training, with the remaining 20% reserved for testing.

### 3.2. Overall Framework Architecture

We denote the labeled dataset from the source domain as $X_{S}={\left\{x_{s}^{i}\right\}}_{i=1}^{N_{s}}$ and the unlabeled dataset from the target domain as $X_{T}={\left\{x_{t}^{i}\right\}}_{i=1}^{N_{t}}$, where $X_{S}={\left\{x_{s}^{i}\right\}}_{i=1}^{N_{s}}$ has a gold standard partition $Y_{S}={\left\{y_{s}^{i}\right\}}_{i=1}^{N_{s}}$. They were both collected randomly from both domains. Here, $N_{s}$ represents the quantity of data in the source domain, while $N_{t}$ denotes the quantity of data in the target domain. For simplicity, the proposed framework is denoted as FACPS, an abbreviation for feature alignment and cross-pseudo-supervision.

The overall framework proposed is illustrated in Figure 2, which provides an overview of our framework and consists of two parts. The first part is the Cross-Modal Feature Alignment Sub-Network, comprising a shared encoder E and two domain-specific decoders, $D_{s}$ and $D_{t}$. The shared encoder and domain-specific decoders are utilized for image reconstruction and cross-modal image generation. We align feature distributions in both directions using the Cross-Modal Feature Alignment Sub-Network to ensure that the synthesized images maintain their original structures via employing a consistency loss method [43]. The second part is the Pseudo-Supervised Dual-Stream Segmentation Sub-Network, which is composed of two identical segmentation sub-networks. The prediction of one network serves as pseudo-labels to supervise the optimization process of the other network. Due to the data’s diverse origins and significant span, there exist non-overlapping distributions, making direct supervision prone to training instability. To stabilize the training process and prevent collapse, we propose the Cross-Domain Distance Perception Module, significantly improving the quality of supervised labels based on cross-domain distances. In the following sections, we will provide detailed descriptions of our method from these two perspectives.

### 3.3. Cross-Modality Feature Alignment Sub-Network

In order to achieve better feature alignment, we developed a novel feature alignment network, where two translation sub-networks are dedicated to transforming images from the source domain to the target domain and vice versa. These networks are based on adversarial losses and incorporate reconstruction losses to better preserve structural information. Additionally, we share the encoder network between the Feature Alignment Sub-Network and the Segmentation Sub-Network, facilitating feature alignment. This approach differs from the methods proposed by Chen et al. [8] and Han et al. [13]. Chen et al. first mapped images to common feature space and then aligned feature distributions through decoders. Han et al. focused on distinguishing between the data originating from the target domain and the source domain to enforce bi-directional feature alignment. In contrast to these methods, our approach emphasizes expressing domain-specific forms through independent decoders for both the source and target domains, thereby compelling the shared encoder to learn domain-invariant feature representations exclusively.

As shown in Figure 2, the feature alignment network consists of a shared encoder (*E*) and a domain-specific decoder (*G*). The encoder maps the source image ($x_{s}$) to a latent space. Then, the target decoder ($G_{t}$) maps the features from the potential space to the target domain to generate a fake target domain image ($x_{s\to t}$) and tries to deceive the target discriminator ($D_{t}$), which is responsible for determining the authenticity of the fake target domain image ($x_{s\to t}$) and the real target domain image ($x_{t}$). $D_{t}$ is updated through the adversarial loss $L_{adv}^{t}$ as

(1) $$\begin{array}{cc} L_{adv}^{t}(E,G_{t},D_{t})= & E_{x^{t}∼X^{t}}\left[\log D_{t}\left(x^{t}\right)\right]\\ \; & +E_{x^{s}∼X^{s}}\left[\log\left(1-D_{t}\left(G_{t}\left(E\left(x^{s}\right)\right)\right)\right)\right]. \end{array}$$

Here, $D_{t}$ needs to maximize $L_{adv}^{t}$ so that it can recognize real or fake images, while *E* tries to minimize $L_{adv}^{t}$ so that $x_{s\to t}$ looks like the real target image. To ensure the effectiveness of the encoder and decoder and the content of the source-domain image remains unchanged, and we performed a reverse image transformation on the generated fake image and reconstructed the original input image as $x_{s\to s}$ and $x_{t\to t}$. Specifically, we obtained $x_{s\to s}$ = $G_{s}\left(E\left(x^{s}\right)\right)$ and $x_{t\to t}$ = $G_{t}\left(E\left(x^{t}\right)\right)$. The reconstructed image should be consistent with the original image; therefore, we can obtain a consistency loss for the target domain.

(2) $$L_{recon}^{t}\left(E,G_{t}\right)=E_{x^{t}∼X^{t}}{\left\|G_{t}\left(E\left(x^{t}\right)\right)-x^{t}\right\|}_{1}.$$

Similarly, the source-domain Discriminator $D_{s}$ also performs corresponding operations to better convert $x_{t}$ to a source-like image. Additionally, we conducted inverse image transformations on the generated fake images and reconstructed the original input images. Specifically, we obtained adversarial losses and cycle consistency losses as follows:

(3) $$\begin{array}{c} L_{adv}^{s}\left(E,G_{s},D_{s}\right)=E_{x^{s}∼X^{s}}\left[\log D_{s}\left(x^{s}\right)\right]\\ +E_{x^{t}∼X^{t}}[\log\left(1-D_{s}\left(G_{s}\left(E\left(x^{t}\right)\right)\right)\right))], \end{array}$$

(4) $$L_{\mathrm{recon}}^{s}\left(E,G_{s}\right)=E_{x^{\prime }∼X}{∥G_{s}\left(E\left(x^{s}\right)\right)-x^{s}∥}_{1}.$$

Up to this point, we obtained the optimization losses for the shared encoder and domain-specific decoders in both the target domain and the source domain. The computation formulas are as follows:

(5) $$\begin{array}{cc} L_{gen}^{t} & =\lambda_{recon}L_{recon}^{t}+\lambda_{adv}L_{adv}^{t},\\ L_{gen}^{s} & =\lambda_{recon}L_{recon}^{s}+\lambda_{adv}L_{adv}^{s}, \end{array}$$

where $\lambda_{\mathrm{recon}}$ and $\lambda_{adv}$ are hyperparameters used to adjust the weights of each loss term. In our experiments, the corresponding values were set to 1.0 and 0.02, respectively.

#### Self-Attention Module

**Figure 3:**
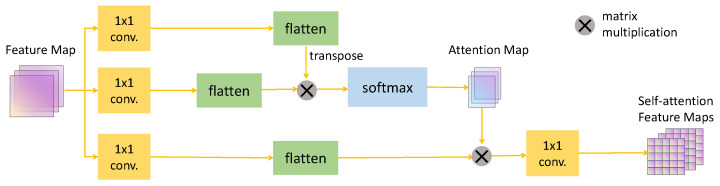
Schematic diagram of the self-attention module.

To effectively model long-range dependencies within images, inspired by the method proposed by Zhang et al. [15], a self-attention module was introduced. By computing the correlation between two positions in the image, an attention map is generated for each pixel in the image matching the size of the image, which reflects the semantic correlation between this point and other points. For each pixel in the image, the self-attention mechanism encourages the model to pay more attention to regions highly correlated with that pixel. Introducing this semantic correlation not only benefits better feature alignment in the model, but it also promotes the learning efficiency of the segmentation model. Although self-attention increases model complexity and training duration, its computational demand is largely contingent on the input image size. To offset this, we uniformly preprocessed inputs to 256 × 256, thereby minimizing computational overhead. The framework of the self-attention is illustrated in Figure 3. The features from the previous layer $f\in R^{C\times H\times W}$ were first passed through two convolutional kernels $C_{1}$ and $C_{2}$, generating feature maps $C_{1}\left(f\right)$ and $C_{2}\left(f\right)$, respectively. At that stage, it was $\left\{C_{1}\left(f\right),C_{2}\left(f\right)\right\}\in R^{C\times H\times W}$. The feature map was then used as the input to the Softmax layer, and the resulting output was the Attention Map A, which can be expressed as

(6) $$A=Soft\max(C_{1}{\left(f\right)}^{T}C_{2}\left(f\right)).$$

Simultaneously, another feature map $C_{3}\left(f\right)$ was obtained through convolutional kernel $C_{3}$. Then, following the same operations as described above, the features were reshaped and multiplied with the attention map calculated using the formula mentioned earlier. The resulting matrix was then passed through the final convolutional kernel $C_{4}$ to obtain the self-attention feature map $f_{att}$, which can be expressed as

(7) $$\begin{array}{cccc} & & f_{att}=C_{4}\left(AC_{3}\left(f\right)\right). & \end{array}$$

Finally, by performing a residual connection between the output of the self-attention mechanism and the input features, we obtained the final output, which is represented as

(8) $$\begin{array}{cccc} & & f_{att}=\lambda_{att}f_{att}+f, & \end{array}$$

where $\lambda_{att}$ is a learnable parameter, initially initialized to 0, guiding the model to explore local spatial information first, and this is then followed by refinement using the self-attention mechanism.

Following the strategy of Zhang et al. [15], self-attention modules were added to the last convolutional layer of the encoder. Each self-attention module consisted of four convolutional layers, with a kernel size of 1 × 1 and a stride set to 1.

### 3.4. Cross-Pseudo-Supervised Dual-Stream Segmentation Sub-Network

Previous methods based on generative adversarial networks have shown promising results in domain adaptation. However, for cross-modal image segmentation tasks, there are often issues such as poor instance segmentation performance and unstable training. Since we lack labels in the target domain, unsupervised domain adaptation can be considered as semi-supervised learning. Chen et al. [16] proposed a method where the output of one network is used to supervise the training of another network to utilize the unlabeled data in the target modality as much as possible (source domain data can also be considered as unlabeled data). This method is suitable when the data come from the same medical-imaging modality (such as CT) but different centers, with relatively small variations compared to cross-modal differences, thus usually producing high-quality supervision. However, in our task, the data came from different medical-imaging modalities (such as CT and MR), and the significant differences in the training samples from different modalities may have led to low-quality pseudo-labels. In this work, inspired by Yao et al. [44] and Sutter et al. [45], we propose a Cross-Domain Distance Perception Cross Pseudo-Supervision mechanism to improve the quality of labels and reduce the impact of low-quality labels. This mechanism provides cross-domain pseudo-variance by measuring the cross-domain pseudo-distance between the target domain and the source domain, thereby enhancing the supervision quality of pseudo-labels. Specifically, the image similar to $x_{s\to t}$ was passed through a shared encoder and then fed into the Dual-Stream Segmentation Sub-Network S(θ). This segmentation sub-network consists of $S(\theta_{1})$ and $S\left(\theta_{2}\right)$, which have the same structure but different initialization. For each segmentation network, we obtained predictions for the original image $x_{s}$ and the image similar to the target domain $x_{s\to t}$ in $S\left(\theta_{1}\right)$ by

(9) $$\begin{array}{cc} p_{o}^{1} & =S\left(\theta_{1}\right)\left(E(x_{s})\right),\\ p_{c}^{1} & =S\left(\theta_{1}\right)\left(E(x_{s\to t})\right). \end{array}$$

Similarly, we passed the original image $x_{s}$ and the image similar to the target domain $x_{s\to t}$ into the segmentation network $S\left(\theta_{2}\right)$, resulting in

(10) $$\begin{array}{cc} p_{o}^{2} & =S\left(\theta_{2}\right)\left(E(x_{s})\right),\\ p_{c}^{2} & =S\left(\theta_{2}\right)\left(E(x_{s\to t})\right). \end{array}$$

After obtaining predictions $p_{o}^{2}$ and $p_{c}^{2}$ from the original image $x_{s}$ and the transformed target-domain image $x_{s\to t}$, respectively, we calculated the average of $p_{o}^{2}$ and $p_{c}^{2}$ to obtain the ensemble prediction result ${\hat{p}}_{avg}^{1}$:

(11) $$\begin{array}{cc} {\hat{p}}_{avg}^{1} & =[p_{o}^{1}+p_{c}^{1}]/2,\\ {\hat{p}}_{avg}^{2} & =[p_{o}^{2}+p_{c}^{2}]/2. \end{array}$$

Cross-pseudo-supervision is suitable for data that come from the same imaging modality but different centers, where the variation is relatively small compared to inter-modalities. For cross-modal data, such as CT and MRI, the significant differences between training samples from different modalities lead to low-quality pseudo-supervision. Inspired by Yao et al. [44], we propose a cross-domain distance awareness module to enhance cross-pseudo-supervision in order to improve the quality of cross-pseudo-supervision. By quantifying the cross-domain pseudo-distance between the source and target domains, the negative impact of low-quality labels on model training is reduced. If there is a large discrepancy between two predictions, the calculated distance value will also be large, reflecting the relatively low quality of the two predictions. As such, the KL divergence is used to measure the discrepancy between two predictions, and the pseudo-distance can be represented as follows:

(12) $$\begin{array}{c} d_{1}=E\left[p_{c}^{1}log\left(\frac{p_{c}^{1}}{p_{o}^{1}}\right)\right],\\ d_{2}=E\left[p_{c}^{2}log\left(\frac{p_{c}^{2}}{p_{o}^{2}}\right)\right]. \end{array}$$

The cross-pseudo-supervision scheme offers dual advantages. It ensures consistency in predictions from various networks for the same image, targeting low-density decision boundaries. Later, it stabilizes pseudo-segmentation, enhancing accuracy beyond conventional supervised learning. This acts as an effective data expansion, boosting the training quality of the segmentation network. After obtaining the prediction distance from the two networks, we propose the enhanced cross-domain distance-aware loss function $L_{dacps}$. Since the input images are $x_{s}$ and $x_{s\to t}$, we named this loss function $L_{dacps}^{s\to t}$:

(13) $$\begin{array}{cc} L_{dacps}^{s\to t} & =E\left[e^{-v1}Lce\left({\hat{p}}_{avg}^{2},P^{1}\right)+d_{1}\right]+,\\ \; & E\left[e^{-v_{2}}Lce\left({\hat{p}}_{avg}^{1},P^{2}\right)+d_{2}\right], \end{array}$$

where $P^{1}$ and $P^{2}$ are one-hot-encoded pseudo-labels generated from predicted probability maps ${\hat{p}}_{avg}^{1}$ and ${\hat{p}}_{avg}^{2}$; $L_{ce}$ represents the cross-entropy loss; and E represents the mathematical expectation. If the input images are $x_{t}$ and $x_{t\to s}$, then, similarly, the cross-domain distance perception loss $L_{dacps}^{t\to s}$ can be obtained. In the supervised learning part, we used the Dice loss to optimize the segmentation sub-network. The loss function can be represented as follows:

(14) $$\begin{array}{cccc} & & L_{s}=L_{\mathrm{Dice}}\left(p_{o}^{1},y_{s}\right)+L_{\mathrm{Dice}}\left(p_{o}^{2},y_{s}\right). & \end{array}$$

Our method leverages the strengths of CPS—ensuring consistent predictions across networks for a given image and mimicking data expansion. Coupled with the benefits of KL divergence, it suppresses the impact of low-quality pseudo labels, making it robust to the initial quality of these labels, which is applicable to cross-modal tasks.

### 3.5. Total Loss

Combining the discussions from the above two sections, the overall loss function can be represented as follows:

(15) $$L=\lambda_{gen}^{t}L_{gen}^{t}+\lambda_{gen}^{s}L_{gen}^{s}+\lambda_{s}L_{s}+\lambda_{st}L_{dacps}^{s+t}+\lambda_{ts}L_{dacps}^{t\to s},$$

where $\lambda_{\mathrm{gen}}^{t}$, $\lambda_{\mathrm{gen}}^{s}$, $\lambda_{s}$, $\lambda_{st}$, and $\lambda_{ts}$ are hyperparameters balancing the weights of each module loss. In the experiments, each training iteration involved multiple network update steps. Firstly, $L_{gen}^{t}$ was used to generate images similar to the target domain and update the shared parameter encoder E, aligning the feature distributions of the source domain and the target domain while transforming the images. Secondly, $L_{gen}^{s}+L_{s}$ was introduced to generate images similar to the source domain and to begin training the segmentation network, extracting features for segmentation results, and further enforcing deep feature alignment. Finally, the source-domain images, target-domain images, generated target-domain similar images, and source-domain similar images were fed into the Dual-Stream Segmentation Sub-Network. The proposed $L_{docps}^{s\to t}+L_{dacps}^{t\to s}$ was used to optimize the network, improving the quality of pseudo-labels and performing feature alignment again. In our experiments, the corresponding values were set as $\lambda_{gen}^{t}=1.0$, $\lambda_{gen}^{s}=1.0$, $\lambda_{s}=1.0$, $\lambda_{st}=2.0$, and $\lambda_{ts}=2.0$. We used the Adam optimizer, where the learning rate was set for the segmentation sub-networks S(θ), the domain-specific decoders $G_{s}$ and $G_{t}$ was set to $1\times 10^{-3}$, and the learning rate for the shared encoder E, as well as the discriminators $D_{s}$ and $D_{t}$, was set to $2\times 10^{-4}$. During each training iteration, we sequentially updated different modules. In the inference stage, we used the shared parameter encoder E to extract features and then feed them into the segmentation sub-networks. The outputs of the two segmentation sub-networks were used as the final segmentation results.

## 4. Experimental Results and Discussion

### 4.1. Experimental Setup

The experiments were conducted on the Ubuntu 20.04 operating system, with code design and implementation carried out using Python and relevant development packages. The deep-learning framework employed was PyTorch, and the iterations were performed on two NVIDIA RTX 4090 GPUs with a memory size of 48 GB using a batch size of 4. During the data preprocessing stage, each slice was resampled to 256 × 256. To expedite network convergence, Z-score was performed using the method wherein the mean intensity was subtracted and the result was divided by the standard deviation. Subsequently, the data were shifted to the range [−1, 1]. In the training phase of the network, random cropping and rotation augmentation operations were additionally applied. It is worth noting that, for a fair comparison, we did not consider the impact of noise and artifacts, and no denoising algorithms were used during the preprocessing stage.

For the shared parameter encoder of the Feature Alignment Sub-Network, following the construction strategy of Han et al. [13], we used the encoder of Deeplab-ResNet50. For the domain-specific decoders $G_{s}$ and $G_{t}$ of the Feature Alignment Sub-Network, they had the same structure but did not share parameters. Each consisted of three residual blocks and three deconvolution layers. The discriminators $D_{s}$ and $D_{t}$ contained four convolutional layers but did not share parameters. For the Dual-Stream Segmentation Sub-Networks $S(\theta_{1})$ and $S(\theta_{2})$, we implemented two segmentation networks using DeepLabv3+ [46] and ResNet50 [47] as backbones, respectively. The backbone networks were initialized with weights trained on ImageNet [48], while the other layers of the segmentation networks were initialized with random noise.

Our experiments used three evaluation metrics. For the abdominal organ segmentation, the Dice coefficient and Average Surface Distance (ASD) were employed as evaluation metrics. For the brain tumor segmentation, the Dice coefficient and Hausdorff Distance (HD) were utilized as evaluation metrics. Furthermore, to better assess the performance and the stability of model retraining, results were obtained using three different model parameter initializations, and the experimental outcomes were presented in terms of both the mean and standard deviation.

### 4.2. Comparison Results

This section primarily validates the proposed FACPS algorithm on the abdominal multi-organ dataset and the Brats2018 dataset and visualizes the segmentation results. We mainly compared the following methods: (1) Supervised Learning (ST)—a segmentation model that is trained using annotated target domain data, and its results can serve as the upper bound for unsupervised domain adaptation methods. (2) No Adaptation (NA)—a segmentation model that is trained with annotated source domain data, which are then directly applied to this model for target-domain data prediction. (3) Adaoutput [11]—one of the domain adaptation methods that performs an adversarial task between source prediction mapping and target prediction mapping. (4) CycleGAN [43]—initially, a CycleGAN is trained with source- and target-domain data, followed by using CycleGAN for modality transfer from the source domain to the target domain. Subsequently, a segmentation model is trained with the synthetic target domain data and are then directly applied to target-domain data prediction. (5) SIFA [8]—this method aligns the data distribution of the two domains at both feature and image levels, and it is used for cross-modality heart-segmentation tasks. (6) SymDA [13]—this method proposes the idea of deep symmetric adaptation, and it is applicable to multiple medical image segmentation tasks. In addition, to better evaluate the performance, we reported the mean and standard deviation of the three different model initializations.

#### 4.2.1. Results on Abdominal Organ Segmentation

We performed bidirectional domain adaptation for the CT and MR data. For convenience, domain adaptation from MR as the source domain to CT as the target domain was abbreviated as MR-CT; conversely, the CT as the source domain to MR as the target domain was abbreviated as CT-MR. The experimental results are presented in Table 1 and Table 2.

Firstly, in both MR-CT and CT-MR, compared to the Supervised Training (ST) method, the performance of the No Adaptation (NA) method significantly decreased, with the average Dice score dropping by 83.85% and 88.21% and the Average Surface Distance (ASD) coefficient increasing by 51.39 and 52.56, respectively. Particularly in the spleen segmentation, the Dice score decreased by 90.32% and 87.5%. The substantial performance decrease highlights the significant impact of the domain gap between modalities on the segmentation model’s performance, indicating that the NA method essentially loses its segmentation ability when faced with data from an unknown domain. Compared to the NA method, FACPS improved the Dice score by 79.09% and 84.45%, respectively. In comparison with the existing methods, FACPS achieved the best results in the segmentation of four different organs and was the optimal method in both Dice and ASD metrics, proving the effectiveness of the proposed approach.

**Figure 4:**
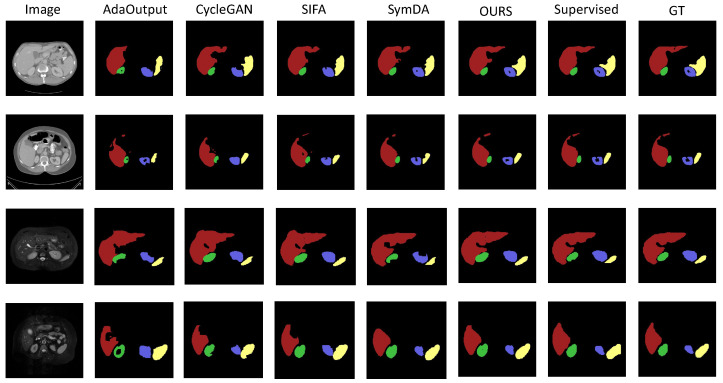
Illustration of the segmentation results by different methods for abdominal CT images (top two rows) and MR images (bottom two rows).

Secondly, FACPS outperformed the existing methods that use feature alignment. As shown in Table 1 and Table 2, the proposed method significantly surpassed CycleGAN by 6.44% and 3.8% in average Dice scores for MR-CT and CT-MR, respectively. This further proves the effectiveness of the proposed method. Moreover, compared to the classical feature-alignment domain adaptation work SIFA, FACPS improved by 3.93% (MR-CT) and 1.45% (CT-MR). For SymDA, the proposed method outperformed by 1.12% in MR-CT. The superior performance of FACPS was primarily due to the introduction of a pseudo-supervision mechanism based on cross-domain distance awareness for training the dual-stream segmentation network, which helps to eliminate the domain gap, thus avoiding poor feature alignment conversion results that affect the segmentation network and allowe the network to absorb more semantic knowledge. Notably, the proposed method showed lower variance in the Dice score than existing methods, indicating more stable network performance. The two-dimensional visualization results comparing FACPS on the abdominal dataset with existing methods are shown in Figure 4.

#### 4.2.2. Results on Brain-Tumor Segmentation

The experimental results on brain tumor segmentation are shown in Table 3. Similar to the abdominal multi-organ dataset, we first discussed the results of the Supervised Training (ST) and No Adaptation (NA) experiments. Due to differences in patterns and distributions, the Dice score decreased by 63.96%, while the Hausdorff Distance (HD) increased by 36.53. All adaptation methods exhibited similar trends on this dataset as on the abdominal multi-organ dataset.

**Figure 5:**
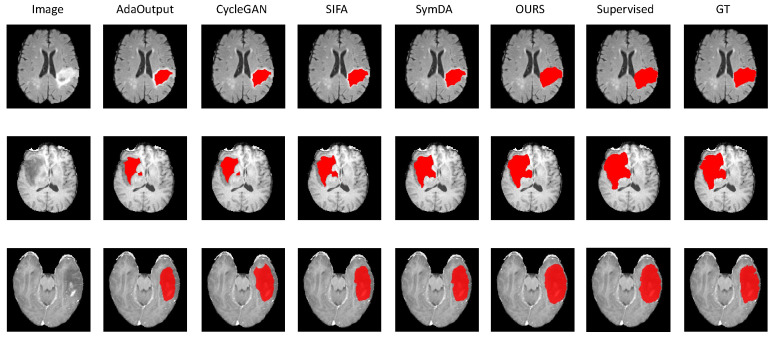
Brain-tumor segmentation results obtained from different methods in the unsupervised domain adaptation task. The T2 modality was used as the source domain and then transferred to the FLAIR, T1, and T1CE modalities (from top to bottom), respectively.

Secondly, compared to NA, our method improved the average Dice score on the T1, T1CE, and FLAIR modalities from 13.11% to 69.18%, as well as reduced the HD from 45.35 mm to 10.47 mm, further validating the effectiveness of the proposed method. The most significant improvement was observed in the FLAIR modality with a Dice increase of 58.86%. The experimental results in Figure 5 demonstrate that accurate prediction of tumors is challenging without domain adaptation. In contrast, the proposed method outperforms existing methods, accurately locating tumor positions across modalities and essentially generating correct segmentation results.

Lastly, all of the methods shared a common characteristic: the Dice score was consistently higher under the FLAIR modality than the other two modalities. This could be because FLAIR is inherently more similar to the source-domain T2 modality, indicating that the degree of modality difference significantly impacts the test results, potentially leading to severe performance degradation in cross-modality segmentation tasks. It is noteworthy that the proposed FACPS method significantly improved performance across both Dice and HD metrics for cross-modality segmentation. At the same time, this method demonstrated smaller performance variance, further verifying the stability of the proposed method in network performance and its resilience against model collapse.

### 4.3. Ablation Study

We performed ablation experiments on the abdominal multi-organ dataset to verify the effect of various constraints on the whole network. In this chapter, we discuss how the proposed FACPS method involves three main modules to enhance the model’s performance, including the feature alignment sub-network, the dual-stream segmentation network with cross-pseudo-supervision, and the self-attention module. To validate their effectiveness individually, an ablation study was conducted using the abdominal multi-organ data for CT-MR domain adaptation. The settings for the ablation study are shown in Table 4, where “✓” indicates the selection of a loss or module. Specifically, the study began with training the segmentation network using only the segmentation loss, denoted as SegOnly; next, the target-domain segmentation loss $L_{st}$ was introduced to validate the effectiveness of the feature alignment network and was denoted as FA; then, the loss $L_{cps}$ was added to validate the effectiveness of the cross-pseudo-supervision mechanism and was denoted as FA+CPS; subsequently, loss $L_{cps}$, which includes both $L_{dacps}^{s\to t}$ and $L_{dacps}^{t\to s}$, was used to validate the effectiveness of the cross-domain distance awareness module and was denoted as FA+DACPS; and, finally, the there was the introduction of the self-attention module, which was abbreviated as FACPS. The results of the ablation study, as shown in Table 5, reveal that, without domain adaptation, the performance of SegOnly is very poor with the lowest average Dice score (i.e., 3.75%).

**FA**: By aligning the source and target domains through one of the feature alignment sub-networks’ two branches and simultaneously transforming the images of both the target and source domains, performance significantly increased by 66.69% compared to SegOnly. This indicates that the feature alignment sub-network is highly beneficial for domain adaptation.

**FA+CPS**: By further employing cross-pseudo-supervision to train the network, the overall feature alignment is enforced and the consistency of the network’s output is constrained. In this way, combining the images generated by the feature alignment sub-network with CPS for network training improved the segmentation network’s performance by 10.85%.

**FA+DACPS**: Introducing the cross-domain pseudo-distance module to optimize the traditional CPS loss effectively avoided potential issues with traditional CPS, thereby further enhancing performance and model stability. As the experimental results show, the domain adaptation capability significantly increased from 81.29% to 88.35%.

**FACPS**: Further incorporating the self-attention module allows the network to learn tht information related to structural consistency, which is beneficial to the learning process of the feature alignment sub-network and enhances the performance of the segmentation network. As shown in the experimental results, the Dice score increased to 89.94%.

### 4.4. Discussion

This study presents the FACPS framework, a robust approach to cross-modal, medical-image segmentation through enhanced feature alignment and cross-pseudo-supervision learning. Our method demonstrates significant improvements in segmentation precision when adapting from one domain to another, as evidenced by the substantial increase in Dice scores and the reduction in ASD and HD measures across various organ-segmentation tasks. Despite the promising results, we identified several areas for future enhancement when primarily focusing on adapting to the unique challenges of datasets and optimizing computational performance.

Regarding dataset challenges, these include issues with small datasets, class imbalance, and data diversity. To address the challenge of small datasets, we will employ advanced data augmentation and meta-learning strategies to enhance our model’s generalizability and robustness against data scarcity. For class imbalance, we will explore specialized techniques to balance class representation during the training process, ensuring equitable model learning and consistent performance across various classes. Additionally, our method’s effectiveness on multi-organ and brain-tumor segmentation tasks suggests a promising potential for generalization across various datasets. To ensure diversity in datasets, we plan to expand our experiments to encompass a broader range of imaging modalities and conditions, thereby better reflecting the breadth of clinical scenarios and enhancing our model’s adaptability.

In terms of computational efficiency optimization, especially for edge devices, our model’s current evaluation on a 4090 GPU indicates an average case prediction time of 2.896 s, which may still be considerable for devices with less computational power. To this end, we are prioritizing optimizations that will expedite our model’s inference speed for real-time applications without compromising accuracy. This includes investigating model compression techniques and optimizing the inference pipeline.

## 5. Conclusions

This paper introduces a domain-adaptation framework for cross-modal, medical-image segmentation named FACPS. Existing feature alignment methods utilize generative adversarial networks for modality conversion and reconstruction of images. However, features sometimes fail to align well, leading to the converted cross-modal images being ineffective for training the segmentation network and potentially introducing erroneous guidance information. Therefore, this paper proposes FACPS, which can better eliminate the domain gap while stabilizing the training process. By measuring the pseudo-distance between the source and target domains, this network effectively improves the quality of pseudo-labels, avoiding the adverse effects of low-quality labels and thereby promoting the learning process of the entire framework. The experiments conducted on the two challenging tasks verified that, after domain adaptation, the medical-image segmentation results in the target domain were significantly improved. This paper introduces a domain-adaptation framework for cross-modal medical-image segmentation named FACPS. Existing feature alignment methods typically utilize generative adversarial networks for modality conversion and image reconstruction. However, challenges arise when features do not align well, resulting in ineffective cross-modal images for training the segmentation network and potentially introducing misleading guidance information. FACPS incorporates a mechanism to measure the pseudo-distance between the source and target domains. By doing so, the network enhances the quality of pseudo-labels, mitigating the negative impact of low-quality labels and facilitating the learning process within the entire framework. The experimental results from two challenging tasks demonstrate that, following domain adaptation, the medical-image segmentation outcomes in the target domain show significant improvement. 

## Figures and Tables

**Table 1:** Comparison of different methods on the abdominal multi-organ dataset. Bold number highlight the best performance.

Abdominal MRI→Abdominal CT										
Method	Dice (%)					ASD (voxel)				
	Liver	R. kidney	L. kidney	Spleen	Average	Liver	R. kidney	L. kidney	Spleen	Average
ST	93.68 ± 3.57	91.74 ± 0.38	91.74 ± 0.18	92.60 ± 0.19	91.83 ± 0.57	1.11 ± 0.12	1.74 ± 0.23	0.92 ± 0.23	1.26 ± 0.04	1.22 ± 0.09
NA	23.43 ± 2.54	5.33 ± 2.20	1.42 ± 0.95	3.09 ± 1.25	7.98 ± 0.27	24.07 ± 2.05	55.64 ± 3.52	60.26 ± 4.19	71.48 ± 2.01	52.61 ± 3.26
AdaOutput [11]	83.56 ± 1.34	80.21 ± 0.77	79.84 ± 1.14	80.71 ± 0.49	80.83 ± 0.59	1.63 ± 0.06	1.21 ± 0.05	1.64 ± 0.16	1.63 ± 0.07	1.53 ± 0.02
CycleGAN [43]	83.19 ± 1.42	78.54 ± 1.62	78.24 ± 1.68	81.19 ± 1.35	80.63 ± 0.58	1.68 ± 0.09	1.22 ± 0.12	1.21 ± 0.05	1.86 ± 0.07	1.50 ± 0.06
SIFA [8]	83.93 ± 0.78	83.15 ± 1.73	84.46 ± 1.45	82.71 ± 0.59	83.14 ± 0.64	1.14 ± 0.06	1.17 ± 0.11	1.47 ± 0.06	1.59 ± 0.06	1.34 ± 0.01
SymDA [13]	87.21 ± 1.06	85.07 ± 0.56	84.52 ± 0.51	86.00 ± 0.02	85.95 ± 0.36	**1.37 ± 0.21**	1.13 ± 0.13	**1.35 ± 0.08**	**1.66 ± 0.15**	**1.38 ± 0.08**
OURS	**89.41 ± 0.18**	**86.26 ± 0.28**	**87.48 ± 0.50**	**85.77 ± 0.47**	**87.07 ± 0.50**	1.74 ± 0.12	**0.89 ± 0.09**	1.71 ± 0.20	2.66 ± 0.22	1.75 ± 0.08

**Table 2:** Comparison of different Methods on the abdominal multi-organ dataset. Bold numbers highlight the best performance.

Abdominal CT→Abdominal MRI										
Method	Dice (%)					ASD (voxel)				
	Liver	R. kidney	L. kidney	Spleen	Average	Liver	R. kidney	L. kidney	Spleen	Average
ST	92.15 ± 0.31	91.50 ± 0.32	91.26 ± 0.19	93.57 ± 0.33	92.04 ± 0.35	1.22 ± 0.06	2.18 ± 0.16	0.91 ± 0.06	0.57 ± 0.05	1.22 ± 0.08
NA	8.45 ± 0.09	0.79 ± 0.33	0.69 ± 0.42	6.07 ± 0.91	3.83 ± 0.43	37.23 ± 0.86	65.68 ± 2.00	56.43 ± 1.13	44.97 ± 2.24	53.78 ± 10.66
AdaOutput [11]	86.25 ± 1.08	82.40 ± 0.53	83.15 ± 0.47	83.87 ± 0.12	83.60 ± 1.87	1.95 ± 0.03	1.62 ± 0.27	3.47 ± 0.38	2.07 ± 0.23	2.35 ± 0.73
CycleGAN [43]	86.24 ± 0.17	83.47 ± 1.25	81.52 ± 1.04	81.47 ± 1.00	84.48 ± 1.45	2.12 ± 0.11	3.38 ± 0.18	2.11 ± 0.19	2.78 ± 0.15	2.54 ± 0.61
SIFA [8]	86.68 ± 0.11	87.45 ± 0.31	86.39 ± 0.19	85.99 ± 0.11	86.83 ± 0.43	1.64 ± 0.12	0.78 ± 0.19	1.54 ± 0.04	2.62 ± 0.25	1.32 ± 0.38
SymDA [13]	89.63 ± 0.31	86.68 ± 0.07	85.56 ± 0.29	88.74 ± 0.13	87.62 ± 2.06	1.58 ± 0.16	1.87 ± 0.08	3.07 ± 0.16	1.94 ± 0.03	2.17 ± 0.67
OURS	**90.92 ± 0.04**	**88.35 ± 0.04**	**87.56 ± 0.03**	**91.21 ± 0.15**	**88.28 ± 1.42**	**1.05 ± 0.03**	**0.90 ± 0.03**	**1.00 ± 0.04**	**0.75 ± 0.18**	**0.98 ± 0.06**

**Table 3:** Comparison of different methods on the Brats2018 dataset. Bold numbers highlight the best performance.

Method	Dice (%)				Hausdorff Distance (mm)			
	T1	FLAIR	T1CE	Average	T1	FLAIR	T1CE	Average
ST	75.47 ± 0.44	85.21 ± 1.01	72.52 ± 0.47	77.07 ± 0.57	9.04 ± 0.41	5.52 ± 0.42	9.90 ± 0.35	8.82 ± 0.38
NA	4.82 ± 1.64	23.42 ± 5.20	13.09 ± 3.04	13.11 ± 3.16	56.05 ± 8.45	29.47 ± 3.27	49.53 ± 7.44	45.35 ± 7.18
AdaOutput [11]	44.39 ± 0.93	61.09 ± 1.06	33.38 ± 2.02	46.62 ± 1.27	24.67 ± 2.00	20.23 ± 1.27	33.13 ± 3.24	26.01 ± 2.16
CycleGAN [43]	36.76 ± 1.44	66.15 ± 1.10	43.26 ± 0.25	48.06 ± 1.11	27.22 ± 2.09	20.31 ± 1.55	23.04 ± 0.84	23.52 ± 1.67
SIFA [8]	52.70 ± 1.13	68.54 ± 2.39	58.53 ± 1.90	59.26 ± 1.87	19.47 ± 1.04	17.15 ± 1.48	15.27 ± 1.71	17.30 ± 1.37
SymDA [13]	57.09 ± 0.76	81.33 ± 0.53	62.08 ± 0.62	66.50 ± 0.63	14.27 ± 0.56	8.63 ± 0.46	13.71 ± 1.34	12.87 ± 0.98
OURS	**60.07 ± 0.23**	**82.28 ± 0.55**	**66.20 ± 0.60**	**69.18 ± 0.46**	**12.95 ± 0.26**	**8.53 ± 0.38**	**10.93 ± 0.95**	**10.47 ± 0.53**

**Table 4:** Ablation experiment settings.

Method	$L_{s}$	$L_{\mathrm{st}}$	$L_{\mathrm{gen}}^{s}$	$L_{\mathrm{gen}}^{t}$	$L_{\mathrm{cps}}$	$L_{\mathrm{dacps}}$	Self_Att
SegOnly	✓						
FA	✓	✓	✓	✓			
FA+CPS	✓	✓	✓	✓	✓		
FA+DACPS	✓	✓	✓	✓		✓	
FACPS	✓	✓	✓	✓		✓	✓

**Table 5:** Experimental results of the ablation study.

Method	Liver	L.Kid	R.Kid	Spleen	Average
SegOnly	8.35	0.96	0.25	5.34	3.75
FA	78.59	68.69	71.22	63.26	70.44
FA+CPS	83.12	79.26	80.08	82.71	81.29
FA+DACPS	90.24	87.07	85.31	90.79	88.35
FACPS	92.89	87.52	88.31	91.03	89.94

## Data Availability

CHAOS dataset can be found at https://chaos.grand-challenge.org/, BTCV dataset can be found at https://www.synapse.org/Synapse:syn3193805/wiki/217789 and Brats2018 dataset can be found at https://www.med.upenn.edu/sbia/brats2018/data.html (accessed on 1 July 2024).
